# Lock out SIRT4

**DOI:** 10.7554/eLife.102355

**Published:** 2024-09-13

**Authors:** Kaiqiang Zhao, Zhongjun Zhou

**Affiliations:** 1 School of Biomedical Sciences, University of Hong Kong Hong Kong Hong Kong

**Keywords:** kidney fibrosis, sirtuin 4, TGF-β1, splicing, nuclear translocation, CCN2, Mouse, Other

## Abstract

The accumulation of SIRT4 in the nuclei of kidney cells drives kidney fibrosis, so blocking the movement of this protein could be a potential therapeutic strategy against fibrosis.

**Related research article** Yang G, Xiang J, Yang X, Liu X, Li Y, Li L, Kang L, Liang Z, Yang S. 2024. Nuclear translocation of SIRT4 mediates deacetylation of U2AF2 to modulate renal fibrosis through alternative splicing-mediated upregulation of CCN2. *eLife* **12**:RP98524. doi: 10.7554/eLife.98524.

Chronic kidney disease is estimated to affect over 10% of the global population (Kovesdy, 2022). Its most common feature is kidney fibrosis, which is characterized by the accumulation of extracellular matrix proteins in tissues, causing structural damage that can impair the function of the kidneys (Huang et al., 2023).

Previous work revealed that complex mechanisms and protein mediators drive kidney fibrosis, including the protein TGF-β1, which plays a vital role in promoting the expression of pro-fibrotic genes. TGF-β1 acts through a well-established signaling pathway that eventually activates a set of three transcription factors – Smad2, Smad3 and Smad4 – which collectively move from the cytoplasm to the nucleus where they promote the transcription of pro-fibrotic genes (Meng et al., 2016). The transport of molecules from the cytoplasm to the nucleus occurs continuously in cells throughout the body and must be tightly controlled to prevent aberrant traffic that can drive disease.

However, directly inhibiting TGF-β1 signaling to prevent kidney fibrosis is not an ideal therapeutic strategy due to the potential adverse effects of disrupting other biological processes involving TGF-β1. Therefore, a greater understanding of the mechanisms underlying TGF-β1-induced fibrosis is required to identify alternative approaches. Now, in eLife, Lin Kang, Zhen Liang, Shu Yang and colleagues – including Guangyan Yang, Jiaqing Xiang and Xiaoxiao Yang as joint first authors – report how unusual TGF-β1-driven translocation of the protein Sirtuin 4 into the nucleus contributes to the progression of kidney fibrosis (Yang et al., 2024).

Sirtuin 4 (also known as SIRT4 for short) is normally located in the mitochondria. However, Yang et al. (who are based at the First Affiliated Hospital of Southern University of Science and Technology and Hefei University of Technology) found increased amounts of SIRT4 in the nuclei of kidney cells from patients with chronic kidney disease. Similarly, they also discovered elevated levels of nuclear SIRT4 in the kidneys of mice that had been injured during surgery to induce kidney fibrosis experimentally.

Intriguingly, Yang et al. found that deleting the gene for SIRT4 significantly reduced the extent of kidney fibrosis in mice following an injury; this occurred either globally throughout the body or specifically in the epithelial cells lining tubules in the kidney. Consistent with this finding, overexpressing the gene in the same cells markedly enhanced the extent of kidney fibrosis. Taken together, these results suggest that abnormal nuclear accumulation of SIRT4 contributes to kidney fibrosis.

To investigate the mechanism underlying SIRT4-mediated fibrosis, Yang et al. carried out experiments in human kidney tubule epithelial cells to identify which proteins interact with SIRT4 upon TGF-β1 treatment. Interestingly, SIRT4 interacted with several proteins that localized in the nucleus, including U2AF2 (short for U2 small nuclear RNA auxiliary factor 2). This protein plays an important role in assembling the molecular machinery known as a spliceosome, which removes unnecessary parts of pre-mRNA to form the mature mRNA that is translated into a protein (Zorio and Blumenthal, 1999; Agrawal et al., 2016).

Yang et al. found that TGF-β1 treatment induced SIRT4 to remove acetyl groups from U2AF2. This allowed U2AF2 to splice the pre-mRNA of a pro-fibrotic protein called CCN2 (short for cell communication network 2), causing expression of this protein to increase. Together, these findings identify the SIRT4-U2AF2-CCN2 axis as a novel mechanism of TGF-β1-induced kidney fibrosis (Figure 1).

**Figure 1.**
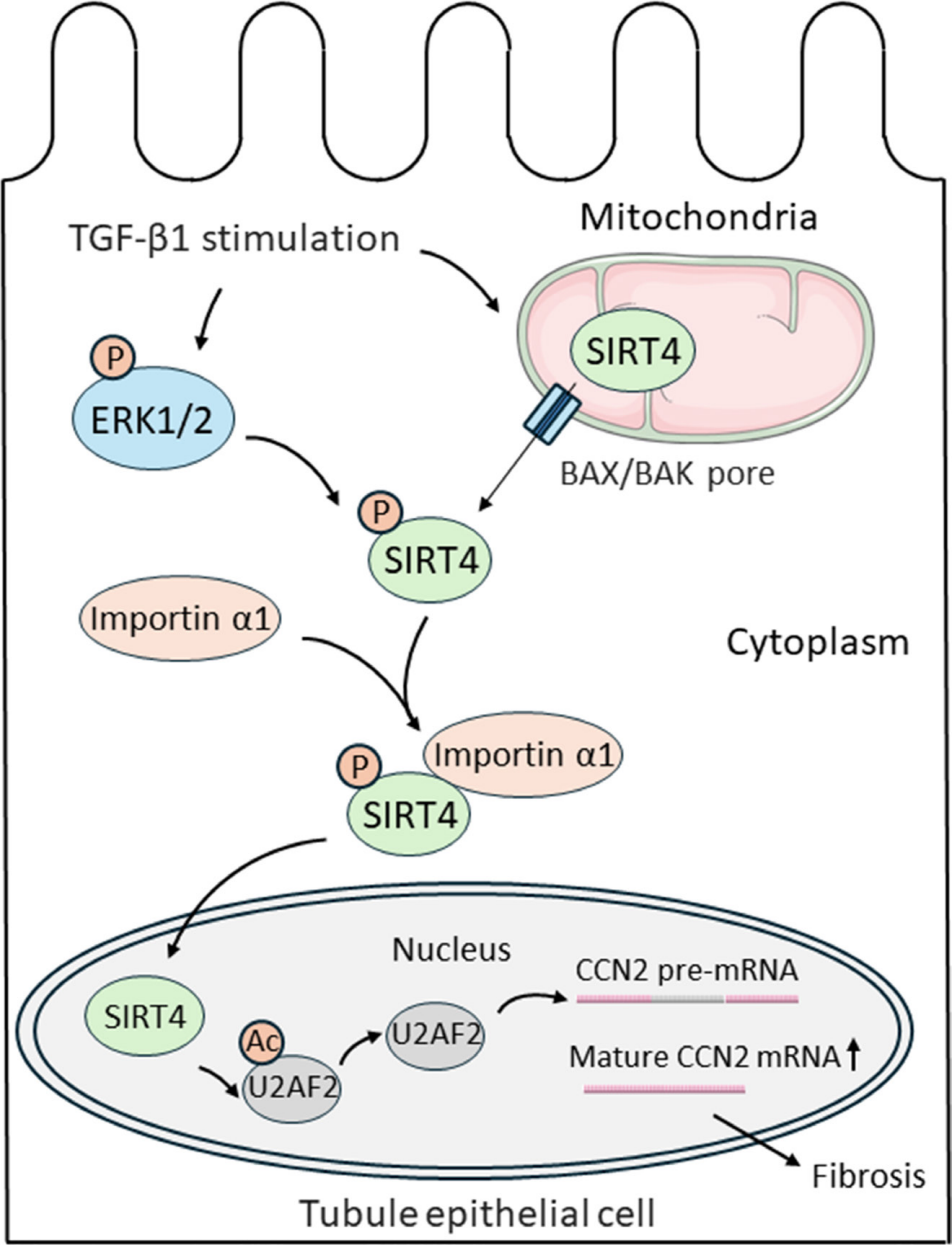
Nuclear import of SIRT4 regulates the progression of kidney fibrosis. TGF-β1 increases the expression of genes that promote kidney fibrosis in epithelial cells lining kidney tubules (black outline). First, TGF-β1 triggers the protein SIRT4 (green) to translocate from the mitochondria (top right) to the cytoplasm through BAX/BAK pores (blue rectangles). Simultaneously, TGF-β1 also activates ERK1/2 (blue oval), which phosphorylate SIRT4 once it is in the cytosol (represented by the addition of an orange circle containing the letter P). Phosphorylated SIRT4 then binds to importin α1 (orange oval) and is transported into the nucleus (bottom) where it removes acetyl groups (orange circle containing the letters Ac) from the protein U2AF2 (grey oval). This allows U2AF2 to splice the pre-mRNA of the pro-fibrotic protein CCN2, resulting in more mature mRNA that can be translated into CCN2 and promote the progression of kidney fibrosis. BAX: B-cell lymphoma 2-associated X protein; BAK: B-cell lymphoma 2 antagonist killer 1 pore; ERK1/2: extracellular signal-regulated kinase 1 and 2; U2AF2: U2 small nuclear RNA auxiliary factor 2; CCN2: cell communication network 2.

Yang et al. next set out to explore how SIRT4 translocates from the mitochondria to the nucleus upon TGF-β1 treatment. The experiments showed that TGF-β1-induced release of SIRT4 from mitochondria occurs through pores formed by protein polymers known as BAX and BAK, which are located in the outer membrane of mitochondria (Cosentino and García-Sáez, 2017). Screening using a variety of protein inhibitors demonstrated that in order to translocate, SIRT4 must be phosphorylated by enzymes known as ERK1 and ERK2. This modification allows SIRT4 to bind to a nuclear-transport receptor called importin α1, which moves the protein into the nucleus. Finally, Yang et al. treated mice with anti-SIRT4 antibodies that only inhibit expression of SIRT4 in the nucleus, and found this targeted disruption could sufficiently alleviate surgery-induced kidney fibrosis.

These findings open an exciting avenue for the development of a novel therapeutic strategy against kidney fibrosis: to lock the ‘bad guy’ – SIRT4 – out of the nuclei of kidney cells. It is conceivable that in the future, small molecules that hinder SIRT4 from interacting with ERK1/2 or importin α1 could be screened or synthesized to treat kidney fibrosis. However, nuclear SIRT4 may play beneficial roles in other cells and tissues (Zeng et al., 2018), thus further clinical studies are needed to evaluate the safety of such a strategy.
